# Porosity Effects on Static Performance of Carbon Nanotube-Reinforced Meta-Nanocomposite Structures

**DOI:** 10.3390/mi14071402

**Published:** 2023-07-09

**Authors:** Farzad Ebrahimi, Ali Dabbagh

**Affiliations:** Department of Mechanical Engineering, Faculty of Engineering, Imam Khomeini International University, Qazvin P.O. Box 3414896818, Iran

**Keywords:** auxetic metamaterials, CNT-reinforced nanocomposites, nonlinear bending, waviness phenomenon

## Abstract

A mixture of outstanding merits of polymer nanocomposites (PNCs) and metamaterials can lead to the development of ultra-light meta-nanomaterials whose high sensitivity can be efficiently used in wearable strain sensors. Thus, reliable data about the performance of structural elements manufactured from such meta-nanomaterials are needed before implementing their design. Motivated by this issue, the negative impacts of pores in the microstructure and carbon nanotubes’ (CNTs’) wavy configuration on the nonlinear bending features of thick beams consisted of auxetic CNT-reinforced (CNTR) polymers are probed for the first time. The impacts of distinct porosity distributions on the mechanical reaction of the system are covered in this article. In addition, a very low computationally cost homogenization is implemented herein to consider the waviness’ influence on the reinforcement mechanism in the auxetic PNC material. Moreover, higher-order shear deformation theory (HSDT) is followed and merged with non-linear definition of strain tensor with the aid of von Kármán’s theory to gather the equations describing the problem. Thereafter, the famous Navier’s exact solution is employed towards solving the problem for thick beams with simple supports at both ends. A comparison of our data with those existing in the literature certifies the accuracy of the presented modeling. The outcomes indicate on the remarkable rise in the flexural deformation of the auxetic PNC beam while the coefficient of porosity is raised. It is also shown that utilization of thick-walled cells in the re-entrant lattice can help to control the system’s total deflection. In addition, if the non-ideal shape of the nanofillers is ignored, the deflection of the meta-nanomaterial beam will be much larger than that of ideal calculations.

## 1. Introduction

The negative Poisson’s ratio satisfied by auxetic arrangement of any desired material avoids shrinkage in such advanced materials [1]. Thanks to this exclusive feature of auxetic metamaterials, bi-directional tension and compression can be observed. Due to this reason, such materials are widely hired by researchers to design and analyze advanced structural elements [2,3]. For example, it was demonstrated that addition of auxetic layers in periodic structures consisting of Aluminum can lead to an increment in the effective modulus of the structure [4]. In another study, it was conceived that composites containing inclusions, whose microstructure possesses an auxetic arrangement, recommend highly negative Poisson’s ratios [5]. It was shown that depending on the shape of the inclusions and hexagonal/hexachiral being of the lattice inside the inclusions, the features of the composite material can be manipulated as desired. Another research study proved that implementation of a re-entrant auxetic lattice instead of non-re-entrant one in the design of honeycomb composites results in extraordinary negative out-of-plane Poisson’s ratios [6]. By the means of a nonlinear continuum modeling, it was concluded that the domain of strain imposed to the hyperelastic auxetic lattice serves a magnificent duty in the approximation of stiffness, Poisson’s ratio, and stress state in the media [7]. Furthermore, experimental reports revealed that gradual change in the material’s lattice from honeycomb to re-entrant can enhance the characteristics of composites strengthened via Kevlar so that the modulus of elasticity reaches to E≃1.53  TPa [8]. On the other hand, it is found that if the corners of hexagonal cells in a honeycomb lattice are replaced with a smaller hexagonal cell and this replacement is repeated, auxeticity can be well observed in the achieved material, also known as hierarchical honeycomb auxetic metamaterials [9]. Numerical simulations indicate on the incredible influence of three-dimensional (3D) auxetic forming of composites on the improvement of the structures’ resistance against blast-type loading [10]. It was also experimentally recognized that formation of soft materials (e.g., polyurethane foam) in an auxetic lattice with the aid of regulating bars increases the modulus of the foam [11]. Such new metamaterials are also reported to be better candidates for energy absorption in mid-range strains [12]. In another endeavor, an improved in-plane modulus and consequently leveled-up buckling resistance for metamaterials is reported by combining rhombic and re-entrant patterns together [13]. It is fascinating to point out that mechanical properties of auxetic metamaterials are easy to change if the geometrical shape and dimensions of the connecting bars in the lattice are changed [14,15]. Analytical investigations proved that the initial value of the auxeticity angle in a re-entrant lattice can tune plastic features of warp and woof auxetic systems [16].

In many studies, mechanical characteristics of systems made of auxetic lattices were monitored. For example, first-order shear deformation theory (FSDT) was employed for investigating the non-linear forced fluctuation characteristics of multi-layered plates containing a core of auxetics [17]. In addition, a zigzag model of plates was chosen to analyze dynamic bending problem of multi-layered continuous systems consisting of an auxetic core that is surrounded by ultra-stiff facesheets [18]. Nonlinear investigation of dynamic responses of auxetic plates was accomplished in Ref. [19] by the means of the third-order shear deformation theory (TSDT) of rectangular plates. In the context of a finite element (FE)-assisted modeling, it was shown that the nonlinear oscillation behaviors of sandwich plates including a 3D auxetic core can be dramatically affected by changing the gradual pattern used for the distribution of the core lattice [20]. In another FE analysis (FEA), it was figured out that if a series of re-entrant lattices of a desired material are attached together so that the auxeticity angle is continuously varied, the capacity of system towards absorbing energy can be enhanced [21]. Moreover, FSDT of doubly-curved shells was employed in [22] for the purpose of probing impact of stiffeners in different types on non-linear externally-stimulated fluctuations of multi-layered shells with auxetic core surrounded by facesheets.

On the other hand, extraordinary properties of nanocomposites have made them excellent choices for the design of advanced structural elements in the recent years [23]. Such ultra-stiff nanomaterials are able to provide big natural frequencies [24,25,26,27], bear against extreme compressive stimulations [28,29,30,31,32,33], and lessen the deflection amplitude [34,35,36,37] of continuous systems. Lately, investigation of the elastic performance of auxetic nanocomposite-made structures has attracted the attention of the academics. In this regard, Kirchhoff plate theorem was implemented in [38] to explore the nonlinear time-varying bending behaviors of auxetic carbon nanotube-reinforced (CNTR) nanocomposite structures. In a group of studies, the nonlinear strains defined by von Kármán are combined with TSDT in order to track the post-buckling paths of auxetic nanocomposite structures reinforced via graphene nanosheets in various environmental conditions [39,40,41,42,43]. Moreover, both theoretical calculation and FE simulation were gathered in [44] toward monitoring the low-velocity impact behaviors of multi-layered beams made from auxetic arrangement of CNTR nanomaterials. Most recently, postbuckling performance of auxetic nanocomposite thin beams was analyzed in the context of an analytical study [45].

Lately, implementation of nanocomposites hosted by a polymeric matrix for strain sensing purposes has been broadly focused [46]. In such cases, it is very important to design sensors able to promote high stretchability and sensitivity. Although such features can be satisfied by polymer nanocomposites as well [46], an improvement in this area can be made by forming the constituent nanomaterial of strain sensors in auxetic lattices [47]. According to [47], gauge factors 24 times bigger than those provided by conventional sensors can be supported if metamaterials are hired. Also, the signal-to-noise ratio of auxetic strain sensors are reported to be about 2.65 times greater than those recommended by conventional ones [47]. Therefore, if a mixture of auxeticity and nanotechnology is employed, ultra-efficient sensors can be designed.

With respect to the above literature review and excellent properties of auxetic nanocomposites, it seems necessary to achieve as more knowledge as possible about bending responses of auxetic nanocomposite structures. To the best of the authors awareness however, such data cannot be found in the existing literature. Thus, the major goal of the current work is to analyze nonlinear bending response of beams consisting of a re-entrant arrangement of CNTR nanocomposites. To this end, a micromechanical homogenization will be carried out to attain the effective properties of an auxetic lattice of nanocomposite by considering the destroying influence of waviness issue on mechanism of stiffness amplification in the constituent nanoengineered material. Afterward, a refined form of Reddy’s TSDT will be combined with the method of energy to formulate the problem by considering the impact of nonlinearities of geometrical kind on the approximation of the continua’s deflection. Once the problem is solved analytically, illustrations will be presented to exhibit the impacts of a sizeable number of variants on the beam’s deflection.

## 2. Theoretical Framework

### 2.1. Homogenization Method

In the current manuscript, an auxetic arrangement of polymer nanocomposites reinforced via CNTs is going to be utilized as the constituent material. To this end, a three-step algorithm will be introduced here. First of all, it will be shown how to find the material properties of meta-nanomaterial if the specifications of porous nanomaterial are in hand. Afterward, we will go through extracting the properties of porous CNTR materials if the equivalent properties of the non-porous one is known. As the final step in this section, revised version of Halpin–Tsai scheme is going to be used to derive the elasticity modulus of non-porous nanocomposite whenever curved nanotubes are used in the manufacturing.

**Figure 1:**
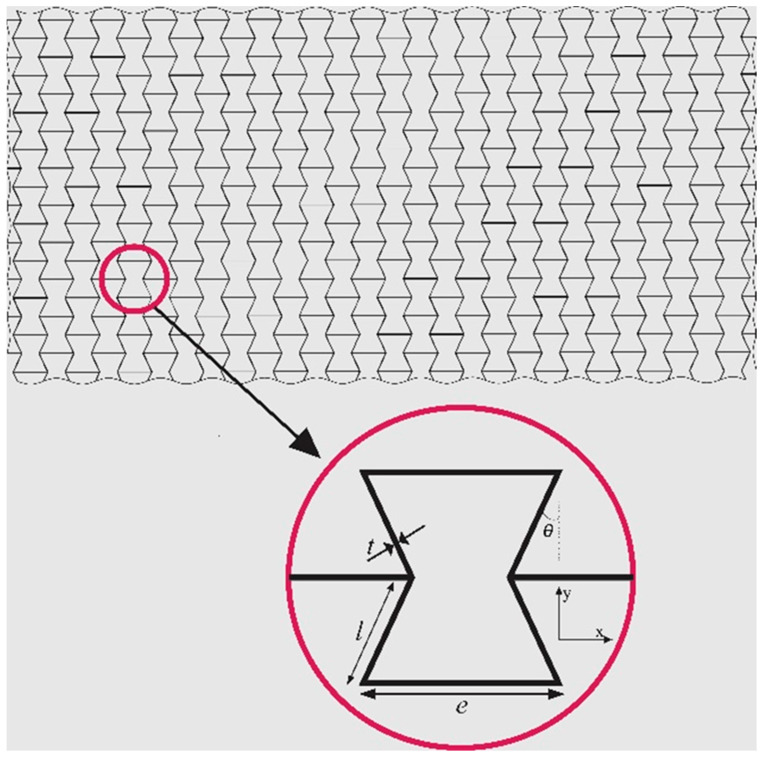
Schematic view of the re-entrant auxetic lattice used in this study. The geometrical parameters involved in determination of the material properties are shown in the zoomed view, too. From A. Dabbagh and F. Ebrahimi [45], Postbuckling analysis of meta-nanocomposite beams by considering the CNTs’ agglomeration, *The European Physical Journal Plus*, 136(11), No. 1168, 2021. Reused with permission from Springer-Nature.

Assume the CNTR polymer nanocomposite to be shaped like a re-entrant lattice. To better understand the geometrical features of the lattice, look at Figure 1. In the zoomed view of this figure, one can simply find the geometrical properties of the auxetic unit cell. The angle θ is known as auxeticity angle. Also, e and l are vertical and inclined lengths of the cell rib, respectively. The cells’ wall thickness of this lattice is shown with t. In order to go through homogenization, a backward scheme will be utilized. To do so, assume the equivalent Young’s moduli and shear moduli of the nanomaterial to be $E_{PNC}$ and $G_{PNC}$, respectively. If polymer nanocomposite is formed in auxetic lattices, the effective modules indicating on the auxetic nanocomposite’s stretch and shear can be calculated as below [17]:

(1) $$E_{eff}=E_{PNC}{\left(t/l\right)}^{3}\frac{\cos\theta }{\left(e/l+\sin\theta \right)\sin^{2}\theta }$$

(2) $$G_{eff}=G_{PNC}\left(t/l\right)\frac{\cos\theta }{\left(e/l+\sin\theta \right)}$$

Now, it is time to extract normal and shear modules of CNTR polymer nanocomposite. In this work, the magnificent impact of essence of voids in the microstructure of the CNTR material is going to be included. Until now, many efforts have been made to account for the porosity effects on the equivalent properties of either homogeneous or heterogeneous materials. For example, the simple rule of the mixture was implemented in some of these attempts to modify the modulus of porous materials [48]. In these studies, a volume fraction was dedicated to the pores and the negative influence of porosity on the stiffness and density of the material was considered by the means of a very simplified mixture’s law. Then, researchers found that modulus of the porous material is related to the density of the same sample in both porous and non-porous states [49,50,51]. This method predicts lower modulus for the material and is more reliable than the simplified rule of the mixture. In none of the above models, the pattern of pores distribution in the material is considered. In this regard, the saturated porous model was hired in some other studies [52,53,54]. Herein, we follow the latter method to approximate the specifications relating to the bulk of nanomaterial if the location of the big and small pores across the thickness is varied. In type-I porosity distribution, bigger pores are located close to the neutral axis; whereas, small pores are adjacent to the upper and lower edges of structure. Porosity type-II however, recommends big pores to be located far from neutral axis. Both of the above distributions are symmetric patterns. In the final distribution profile, pores are assumed to be dispersed in the media, uniformly. According to [23,53], the stiffness and mass density of the porous CNTR nanocomposite can be attained following the following relations:

(3) $$\left\{ \begin{array}{l} E_{PNC}=E_{e}\left[1-e_{1}\cos\left(\frac{\pi z}{h}\right)\right]\\ \rho_{PNC}=\rho_{e}\left[1-e_{m1}\cos\left(\frac{\pi z}{h}\right)\right] \end{array}\right.\;\;\;\;\;,\;\;\;\;\;\mathrm{Porosity}\;\;\mathrm{Type}-I$$

(4) $$\left\{ \begin{array}{l} E_{PNC}=E_{e}\left[1-e_{2}\left\{1-\cos\left(\frac{\pi z}{h}\right)\right\}\right]\\ \rho_{PNC}=\rho_{e}\left[1-e_{m2}\left\{1-\cos\left(\frac{\pi z}{h}\right)\right\}\right] \end{array}\right.\;\;\;\;\;,\;\;\;\;\;\mathrm{Porosity}\;\;\mathrm{Type}-\mathrm{II}$$

(5) $$\left\{ \begin{array}{l} E_{PNC}=E_{e}e_{3}\\ \rho_{PNC}=\rho_{e}e_{m3} \end{array}\right.\;\;\;\;\;,\;\;\;\;\;\mathrm{Porosity}\;\;\mathrm{Type}-\mathrm{III}$$

In the above relations, porosity coefficients corresponding with types I, II, and III of porosity distribution are respectively shown with $e_{1}$, $e_{2}$, and $e_{3}$. Also, $e_{m1}$, $e_{m2}$, and $e_{m3}$ are the mass coefficients of type I, II, and III porous nanomaterials. These coefficients are responsible for modifying the mass density of the porous nanocomposite. It is worth regarding that $E_{e}$ and $\rho_{e}$ indicate on the moduli and density of the non-porous nanocomposite, respectively. It is necessary to mention that the Poisson’s ratio remains unchangeable and cannot be affected by existence of pores in the CNTR nanomaterial (i.e., $\nu_{PNC}=\nu_{e}$). With the aid of the above relations and by considering the relationship between tangent moduli, Poisson’s ratio, and shear moduli in linearly elastic solids of isotropic kind, the shear modulus of porous nanocomposite can be simply found by using $G_{PNC}=E_{PNC}/2\left(1+\nu_{PNC}\right)$.

It must be noticed that only porosity coefficient *e*_1_ is known to us and other coefficients must be determined based on it. These coefficients can be determined by considering the fact that the porosity type cannot affect the total mass of the porous nanomaterial. Thus, below identity is written [23,53]:

(6) $$\int_{0}^{h/2}\sqrt{1-e_{1}\cos\left(\frac{\pi z}{h}\right)}dz=\int_{0}^{h/2}\sqrt{1-e_{2}\left[1-\cos\left(\frac{\pi z}{h}\right)\right]}dz=\int_{0}^{h/2}\sqrt{e_{3}}dz$$

In addition, the mass coefficients of each type of porosity distribution can be related to its corresponding porosity coefficient by the means of following identities [23,53]:

(7) $$1-e_{m1}\cos\left(\frac{\pi z}{h}\right)=\sqrt{1-e_{1}\cos\left(\frac{\pi z}{h}\right)}$$

(8) $$1-e_{m2}\left[1-\cos\left(\frac{\pi z}{h}\right)\right]=\sqrt{1-e_{2}\left[1-\cos\left(\frac{\pi z}{h}\right)\right]}$$

(9) $$e_{m3}=\sqrt{e_{3}}$$

Now, we will derive the features of non-porous CNTR nanocomposites reinforced with non-straight nanofillers. In this investigation, the curved form of the CNTs is covered in the modeling in the framework of a micromechanical scheme. Present model is a revised edition of the Halpin–Tsai algorithm. A waviness coefficient will be introduced in this method to account for the curvy configuration of the nanotubes on the modulus of the nanomaterial. Moreover, the randomized alignment of the wavy CNTs in the polymer is included, too. In addition to all of these merits, it is interesting to mention that complexities existing in previously introduced methods [55,56,57] cannot be seen in this computationally low-cost algorithm. Based upon this approach [58], the extensional moduli of the polymer nanocomposite is calculated via:

(10) $$E_{e}=\frac{1+C\eta V_{r}}{1-\eta V_{r}}E_{m}$$

in which

(11) $$\eta =\frac{C_{w}\left[\alpha E_{CNT}/E_{m}\right]-1}{C_{w}\left[\alpha E_{CNT}/E_{m}\right]+C}$$

In the above relations, the CNT loading will be controlled with CNTs’ volume fraction $V_{r}$. $E_{m}$ and $E_{CNT}$, respectively, denote elastic moduli of polymer and CNT. Also, the geometrical shape of the reinforcing nanofillers is captured in this model with coefficient C defined to be two times of the length of the ideal CNTs divided by their diameter ($C=2l_{CNT}/d_{CNT}$). Because of the fact that length of the reinforcing elements is too many times smaller than the beam thickness, the orientation factor is required to be set on α=1/6 [58]. The impact of curvy form of the CNTs on the Young’s modulus of nanocomposite will be tracked with waviness coefficient $C_{w}$. This coefficient is allowed to vary between zero and one and its utmost value corresponds with ideal CNTs with a straight shape. It must be declared that although this method is very simple, it possesses a high precision in low loadings of nanofillers. This claim was proven in Ref. [58] by comparing the variations in modulus of elasticity against CNTs loading in matrix achieved from this modeling with those gathered from experimental measurements. The results of this comparison can be found in Figure 9 of Ref. [58]. According to this figure, it is conceived that if the waviness coefficient is assumed to be within the range of $0.3<C_{w}<0.4$, the approximated modulus will be very close to those obtained from experiments.

As the final hint, it is worth mentioning that the mass density and Poisson’s ratio and of the non-porous nanocomposite can be calculated via the modified Halpin–Tsai method as follows:

(12) $$\nu_{e}=\nu_{CNT}V_{r}+\nu_{m}\left(1-V_{r}\right)$$

(13) $$\rho_{e}=\rho_{CNT}V_{r}+\rho_{m}\left(1-V_{r}\right)$$

Therefore, the shear modulus can be computed simply following the relation $G_{e}=E_{e}/2\left(1+\nu_{e}\right)$.

**Figure 2:**
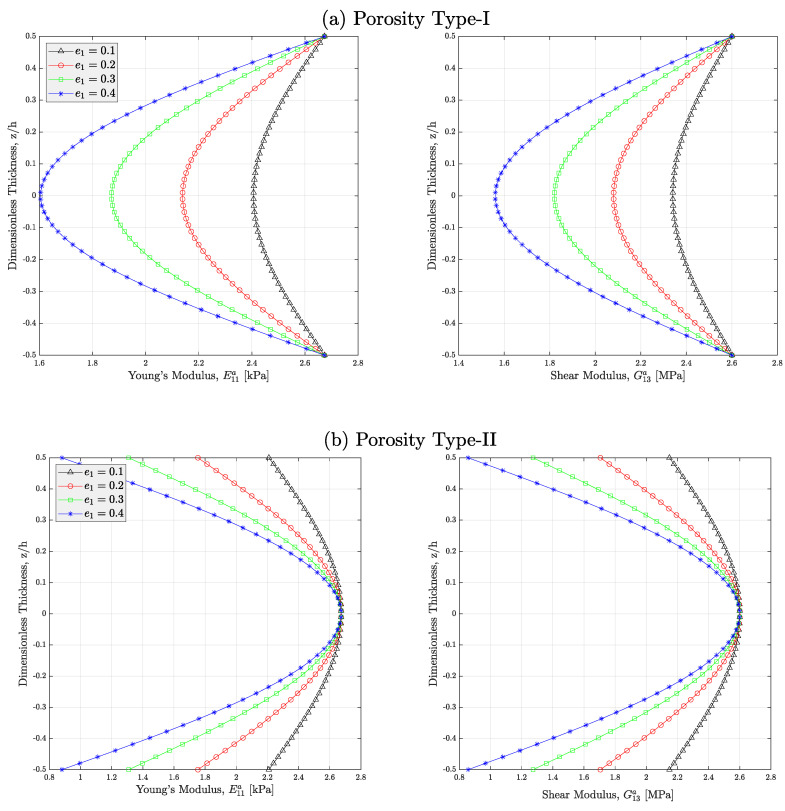

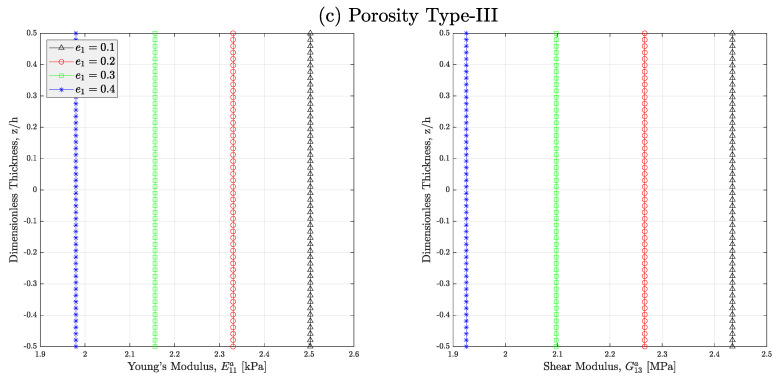
Effect of porosity coefficient *e*_1_ on the variation in Young’s modulus and shear modulus of the auxetic PNC materials with (**a**) type-I, (**b**) type-II, and (**c**) type-III (*V_r_* = 1%, *C_w_* = 0.35).

The impacts of both distribution and coefficient of porosity on the approximation of elasticity and shear modules of the auxetic CNTR nanocomposites are depicted in Figure 2. According to this figure, it can be easily found that both normal and torsional stiffnesses of the auxetic nanomaterial will be lessened once another value, greater in magnitude, is allocated to the porosity coefficient $e_{1}$. Based on this illustration, the worst condition belongs to auxetic nanocomposites with pores distributed across the thickness via type-II. In such porous meta-nanomaterials, the stiffness of the material will be in its minimum value in upper and lower edges of continua. Due to the fact that the distance of such axes from neutral axis is big, those parts serve magnificent duties in the calculation of the cross-sectional rigidities of the continua. That modulus variation differs for each of the mentioned porosity distributions possesses a logical reason. In type-I porous meta-nanocomposites, pore concentration is at the central region across the thickness. Therefore, the minimum stiffness occurs when the dimensionless thickness is zero. In type-II porous nanomaterials, pores are more positioned in near-edge regions and this makes such zones less stiff. Obviously, because the homogeneous distribution of pores is modeled in type-III, all parts of the nanomaterial manifest similar modulus. Therefore, it is predicted that type-II porous auxetic nanocomposites experience greater deflections followed by type-III and type-I porous meta-nanomaterials, respectively.

### 2.2. Problem Kinematics

Herein, the mathematical relations of the structure’s motion are going to be derived. The structure is a beam possessing a rectangular-kind cross-section whose length, width, and thickness are respectively shown with L, b, and h. In this article, we are about to analyze the mechanical behaviors of thick beams whose slenderness ratio is small. In thin-type structures, the influences of shear-induced deflection on the elastic characteristics of the system can be dismissed [59,60,61]. However, this assumption leads to precision reduction if the response of a thick-type beam is going to be monitored. A large number of HSDTs are known by now among which the TSDT of Reddy [62] is the most famous one. In the conventional form of TSDT of beams, three field variables are implemented indicating on the longitudinal deformation, bending deflection, and cross-section’s distortion. Here, refined version of this theory will be utilized that contains an axial displacement as well as two separated variables denoting bending and shear deflections. According to this theorem, the displacement field of the beam can be expressed as below [60]:

(14) $$\begin{array}{l} u_{x}(x,z)=u(x)-\left(z-\tilde{z}\right)\frac{\partial w_{b}(x)}{\partial x}-\left(f(z)-\hat{z}\right)\frac{\partial w_{s}(x)}{\partial x},\\ u_{z}(x,z)=w_{b}(x)+w_{s}(x) \end{array}$$

where u(x) stands for the longitudinal deformation of the beam’s neutral axis; while, $w_{b}(x)$ and $w_{s}(x)$, respectively, denote deflections caused by bending and shear. Also, we have:

(15) $$\tilde{z}=\frac{\int_{-h/2}^{h/2}zE_{eff}dz}{\int_{-h/2}^{h/2}E_{eff}dz},\;\;\;\;\;\;\;\;\;\;\hat{z}=\frac{\int_{-h/2}^{h/2}f(z)E_{eff}dz}{\int_{-h/2}^{h/2}E_{eff}dz}$$

It is clear that the shape function that is in charge of consideration of the profiles of shear stress and strain across the flexural direction is $f(z)=z-4z^{3}/3h^{2}$ according to [62]. Now, the introduced field in Equation (14) can be implemented to extract identities promoting the structure’s normal and shear strain while the influences of von Kármán kind geometrical nonlinearity on beam’s motion is captured. The following strain–displacement relations can be written for the shear deformable beam:

(16) $$\begin{array}{l} \varepsilon_{xx}=\frac{\partial u}{\partial x}+\frac{1}{2}{\left(\frac{\partial (w_{b}+w_{s})}{\partial x}\right)}^{2}-\left(z-\tilde{z}\right)\frac{\partial^{2}w_{b}}{\partial x^{2}}-\left(f(z)-\hat{z}\right)\frac{\partial^{2}w_{s}}{\partial x^{2}},\\ \gamma_{xz}=2\varepsilon_{xz}=g(z)\frac{\partial w_{s}}{\partial x} \end{array}$$

For the goal of achieving the motion equations, virtual work’s principle is going to be used in the current paper. This principle says that changes in the entire energy of an arbitrary structure is obligated to be set to zero. In mathematics language [60]:

(17) δ(U+V)=0

in which U and V, respectively, represent beam’s strain energy and work done by external loads. Following the constitutive relations of linearly elastic solids as well as recalling the definitions introduced in Equation (16), *δU* is easily achieved as:

(18) $$\delta U=\int_{0}^{L}\left\{\begin{array}{c} N_{xx}\left[\frac{\partial \delta u}{\partial x}+\frac{\partial (w_{b}+w_{s})}{\partial x}\frac{\partial \delta (w_{b}+w_{s})}{\partial x}\right]\\ -M_{xx}^{b}\frac{\partial^{2}\delta w_{b}}{\partial x^{2}}-M_{xx}^{s}\frac{\partial^{2}\delta w_{s}}{\partial x^{2}}+Q_{xz}\frac{\partial \delta w_{s}}{\partial x} \end{array}\right\}dx$$

The stress resultants mentioned above are expressed as follows:

(19) $$\left[\begin{array}{l} N_{xx}\\ M_{xx}^{b}\\ M_{xx}^{s} \end{array}\right]=\int_{A}\left[\begin{array}{c} 1\\ z-\bar{z}\\ f(z)-\tilde{z} \end{array}\right]\sigma_{xx}dA,\;\;\;\;\;\;\;\;\;\;Q_{xz}=\int_{A}g(z)\sigma_{xz}dA$$

At the moment, the variation in work performed on the system is going to be formulated. Assume a uniform bending force q functioning on the top edge of the beam. Therefore, we have [60]:

(20) $$\delta V=\int_{0}^{L}q\delta (w_{b}+w_{s})dx$$

Now, if Equations (18) and (20) are inserted in Equation (17), the following motion relations are enhanced:

(21) $$\frac{\partial N_{xx}}{\partial x}=0$$

(22) $$\frac{\partial }{\partial x}\left\{N_{xx}\frac{\partial (w_{b}+w_{s})}{\partial x}\right\}-\frac{\partial^{2}M_{xx}^{b}}{\partial x^{2}}+q=0$$

(23) $$\frac{\partial }{\partial x}\left\{N_{xx}\frac{\partial (w_{b}+w_{s})}{\partial x}\right\}-\frac{\partial^{2}M_{xx}^{s}}{\partial x^{2}}+\frac{\partial Q_{xz}}{\partial x}+q=0$$

### 2.3. Governing Equations

In the former parts, the relations governing the beam’s motion were gathered. In this section, we are about to seek other relations describing the constitutive characteristics of beam kind structures. According to Hook’s law, below 1D identities for the meta-nanocomposite system are:

(24) $$\sigma_{xx}=E_{eff}\varepsilon_{xx},\;\;\;\;\;\;\;\;\;\;\sigma_{xz}=G_{eff}\gamma_{xz}$$

By integrating above relations over the cross-section of structure and recalling Equation (19), the following expressions are enhanced:

(25) $$N_{xx}=A_{xx}\left[\frac{\partial u}{\partial x}+\frac{1}{2}{\left(\frac{\partial (w_{b}+w_{s})}{\partial x}\right)}^{2}\right]$$

(26) $$M_{xx}^{b}=-D_{xx}\frac{\partial^{2}w_{b}}{\partial x^{2}}-D_{xx}^{s}\frac{\partial^{2}w_{s}}{\partial x^{2}}$$

(27) $$M_{xx}^{s}=-D_{xx}^{s}\frac{\partial^{2}w_{b}}{\partial x^{2}}-H_{xx}^{s}\frac{\partial^{2}w_{s}}{\partial x^{2}}$$

(28) $$Q_{xz}=A_{xz}\frac{\partial w_{s}}{\partial x}$$

In the above relations, the terms with integrands containing odd powers of z do not exist due to accurate consideration of neutral axis’s position in Equation (15). The existing cross-sectional rigidities of the beam-type element are easy to calculate using the below definition:

(29) $$\left[\begin{array}{l} A_{xx}\\ D_{xx}\\ D_{xx}^{s}\\ H_{xx}^{s} \end{array}\right]=\int_{A}\left[\begin{array}{c} 1\\ {\left(z-\bar{z}\right)}^{2}\\ \left(z-\bar{z}\right)\left(f(z)-\tilde{z}\right)\\ {\left(f(z)-\tilde{z}\right)}^{2} \end{array}\right]E_{eff}dA,\;\;\;\;\;\;\;\;\;\;A_{xz}=\int_{A}g^{2}(z)G_{eff}dA$$

Now, glancing at Equation (21) reveals that $N_{xx}=\mathrm{cte}$. So, Equations (22) and (23) will be re-formed as follows:

(30) $$N_{xx}\frac{\partial^{2}(w_{b}+w_{s})}{\partial x^{2}}-\frac{\partial^{2}M_{xx}^{b}}{\partial x^{2}}+q=0$$

(31) $$N_{xx}\frac{\partial^{2}(w_{b}+w_{s})}{\partial x^{2}}-\frac{\partial^{2}M_{xx}^{s}}{\partial x^{2}}+\frac{\partial Q_{xz}}{\partial x}+q=0$$

If Equations (25)–(28) are inserted into Equations (30) and (31), the below relations will be attained:

(32) $$A_{xx}\left[\frac{\partial u}{\partial x}+\frac{1}{2}{\left(\frac{\partial (w_{b}+w_{s})}{\partial x}\right)}^{2}\right]\frac{\partial^{2}(w_{b}+w_{s})}{\partial x^{2}}+D_{xx}\frac{\partial^{4}w_{b}}{\partial x^{4}}+D_{xx}^{s}\frac{\partial^{4}w_{s}}{\partial x^{4}}+q=0$$

(33) $$A_{xx}\left[\frac{\partial u}{\partial x}+\frac{1}{2}{\left(\frac{\partial (w_{b}+w_{s})}{\partial x}\right)}^{2}\right]\frac{\partial^{2}(w_{b}+w_{s})}{\partial x^{2}}+D_{xx}^{s}\frac{\partial^{4}w_{b}}{\partial x^{4}}+H_{xx}^{s}\frac{\partial^{4}w_{s}}{\partial x^{4}}+A_{xz}\frac{\partial^{2}w_{s}}{\partial x^{2}}+q=0$$

The above relations describe the beam’s motion. By assuming the unchangeable magnitude of the normal load to be c, it gives:

(34) $$\frac{\partial u}{\partial x}=\frac{c}{A_{xx}}-\frac{1}{2}{\left(\frac{\partial (w_{b}+w_{s})}{\partial x}\right)}^{2}\to u(x)=\frac{cx}{A_{xx}}-\frac{1}{2}\int_{0}^{x}{\left(\frac{\partial (w_{b}+w_{s})}{\partial x}\right)}^{2}dx+d$$

in which d is an unknown that can be attained by the means of the boundary conditions (BCs). Since the beam is regarded to be simply supported, we have:

(35) u(0)=0→d=0

(36) $$u(L)=0\to c=\frac{A_{xx}}{2L}\int_{0}^{L}{\left(\frac{\partial (w_{b}+w_{s})}{\partial x}\right)}^{2}dx$$

According to the above results, one can deduce that:

(37) $$\frac{\partial u}{\partial x}+\frac{1}{2}{\left(\frac{\partial (w_{b}+w_{s})}{\partial x}\right)}^{2}=\frac{1}{2L}\int_{0}^{L}{\left(\frac{\partial (w_{b}+w_{s})}{\partial x}\right)}^{2}dx$$

Substitution of the above identity in Equations (32) and (33) reveals:

(38) $$\frac{A_{xx}}{2L}\int_{0}^{L}{\left(\frac{\partial (w_{b}+w_{s})}{\partial x}\right)}^{2}dx\frac{\partial^{2}(w_{b}+w_{s})}{\partial x^{2}}+D_{xx}\frac{\partial^{4}w_{b}}{\partial x^{4}}+D_{xx}^{s}\frac{\partial^{4}w_{s}}{\partial x^{4}}+q=0$$

(39) $$\frac{A_{xx}}{2L}\int_{0}^{L}{\left(\frac{\partial (w_{b}+w_{s})}{\partial x}\right)}^{2}dx\frac{\partial^{2}(w_{b}+w_{s})}{\partial x^{2}}+D_{xx}^{s}\frac{\partial^{4}w_{b}}{\partial x^{4}}+H_{xx}^{s}\frac{\partial^{4}w_{s}}{\partial x^{4}}+A_{xz}\frac{\partial^{2}w_{s}}{\partial x^{2}}+q=0$$

## 3. Solution Procedure

In the current section of this manuscript, it is attempted to untangle the non-linear deformation problem of auxetic nanocomposite beams. Until now, many numerical and analytical methods have been implemented by researchers to analyze mechanical responses of nonhomogeneous structures [63,64,65,66,67]. Herein, the famous Navier’s algorithm is going to be employed to resolve the problem. According to this approach [60], the bending and shear deflections of a fully simply supported structure is considered to be in the below series form:

(40) $$\left\{\begin{array}{l} w_{b}(x)\\ w_{s}(x) \end{array}\right\}=\sum_{n=1}^{\infty }\left\{\begin{array}{l} W_{bn}\\ W_{sn} \end{array}\right\}\sin\lambda x,\;\;\;\;\;\;\;\;\;\;\lambda =\frac{n\pi }{L}$$

where n is axial mode number; $W_{bn}$ and $W_{sn}$ are amplitudes of bending and shear deflections corresponding to the *n*th mode, respectively. Also, the inserted loading is assumed to obey from the below expression:

(41) $$q(x)=\sum_{n=1}^{\infty }Q_{n}\sin\lambda x$$

In the above definition, the loading amplitude $Q_{n}$ can be gathered by following the below relation:

(42) $$Q_{n}=\frac{2}{L}\int_{0}^{L}q(x)\sin\lambda xdx$$

In this article, the continua is regarded to be under uniform loading and therefore, the magnitude of the exerted force is determined by:

(43) $$Q_{n}=\frac{4q_{0}}{n\pi }\;\;\;\;\;\;\;\;\;(n=1,3,5,\cdots )$$

Once Equations (40) and (41) are substituted in Equations (38) and (39), the below coupled nonlinear equations can be achieved:

(44) $$\begin{array}{l} -\frac{A_{xx}}{2L}\sum_{n=1}^{\infty }{\left(W_{bn}+W_{sn}\right)}^{3}\lambda^{4}\int_{0}^{L}\cos^{2}\lambda xdx\sin\lambda x+D_{xx}\sum_{n=1}^{\infty }W_{bn}\lambda^{4}\sin\lambda x\\ +D_{xx}^{s}\sum_{n=1}^{\infty }W_{sn}\lambda^{4}\sin\lambda x+\sum_{n=1}^{\infty }Q_{n}\sin\lambda x=0 \end{array}$$

(45) $$\begin{array}{l} -\frac{A_{xx}}{2L}\sum_{n=1}^{\infty }{\left(W_{bn}+W_{sn}\right)}^{3}\lambda^{4}\int_{0}^{L}\cos^{2}\lambda xdx\sin\lambda x+D_{xx}^{s}\sum_{n=1}^{\infty }W_{bn}\lambda^{4}\sin\lambda x\\ +H_{xx}^{s}\sum_{n=1}^{\infty }W_{sn}\lambda^{4}\sin\lambda x-A_{xz}\sum_{n=1}^{\infty }W_{sn}\lambda^{2}\sin\lambda x+\sum_{n=1}^{\infty }Q_{n}\sin\lambda x=0 \end{array}$$

If mathematical simplifications are performed, the above coupled differential equations can be re-written as below:

(46) $$\sum_{n=1}^{\infty }K_{bbn}^{L}W_{bn}+\sum_{n=1}^{\infty }K_{bsn}^{L}W_{sn}+\sum_{n=1}^{\infty }K_{n}^{NL}{\left(W_{bn}+W_{sn}\right)}^{3}=\sum_{n=1}^{\infty }Q_{n}$$

(47) $$\sum_{n=1}^{\infty }K_{sbn}^{L}W_{bn}+\sum_{n=1}^{\infty }K_{ssn}^{L}W_{sn}+\sum_{n=1}^{\infty }K_{n}^{NL}{\left(W_{bn}+W_{sn}\right)}^{3}=\sum_{n=1}^{\infty }Q_{n}$$

in which $K_{bbn}^{L}$, $K_{bsn}^{L}$, $K_{sbn}^{L}$, and $K_{ssn}^{L}$ are linear stiffnesses corresponding with any desired mode number of bending and shear deflections in first and second equations, respectively. Also, $K_{n}^{NL}$ is the nonlinear stiffness related to any arbitrary mode number. By solving Equations (46) and (47) for $W_{bn}$ and $W_{sn}$ simultaneously, the deflection amplitude at any natural mode will be gathered. Afterward, the summation of the deflections of system’s natural modes gives us the total deflection of the auxetic nanocomposite beam as well.

## 4. Case Studies

A set of illustrative case studies are presented for the goal of analyzing the nonlinear bending response of a shear deformable beam manufactured from auxetic CNTR nanocomposite. In this section, the beam’s slenderness ratio and thickness will be respectively kept on L/h=10 and h=2  mm, unless other values are reported. The main reason of choosing this geometry is that presented modeling is powerful enough to track the nonlinear behaviors of thick structures. In addition, if not mentioned, the vertical-to-inclined length, thickness-to-inclined length, and angle of auxeticity of the cell rib of the lattice are respectively considered to be e/l=4, t/l=0.0138571, and θ=−45. Table 1 reveals the material inputs of resin and CNTs for reference.

In Table 1, the CNTs’ diameter is considered to be $d_{CNT}=1.356\;\;\mathrm{nm}$. It is worth regarding that this value is reported according to the definition $d_{CNT}=a\sqrt{m^{2}+mn+n^{2}}/\pi$; where graphene’s lattice constant is $a=\sqrt{3}a_{C-C}$ ($a_{C-C}$ is the carbon–carbon bond’s length that is identical with 0.142  nm). Variables m and n are the components of the chiral vector of the CNT. Because we use SWCNT(10, 10) in this article, the diameter of the ideal CNT is assumed to be as what is reported in Table 1.

**Figure 3:**
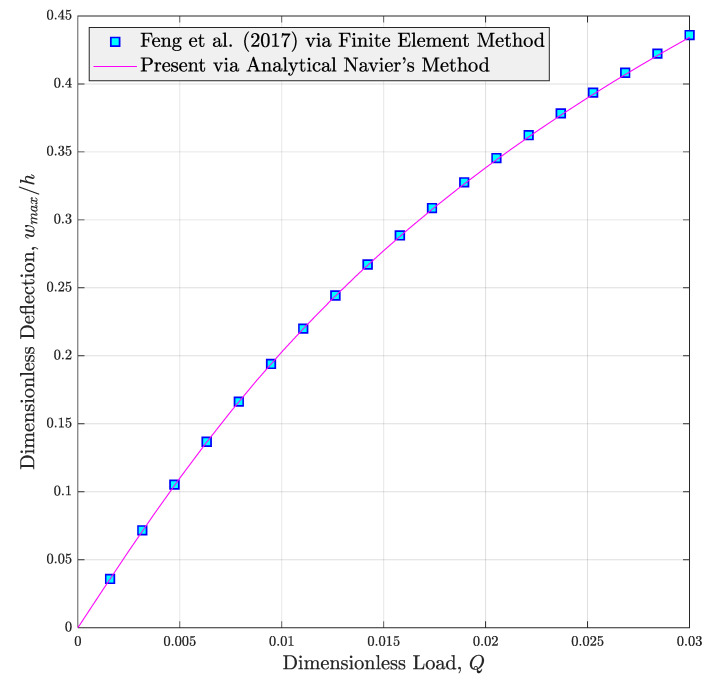
Comparison of the nonlinear bending response of polymer nanocomposite beams reinforced with uniformly distributed GPLs (*f_i_* = 0.5%, *N* = 10, *L*/*h* = 20) [34].

In what follows, the dimensionless form of the beam’s deflection and loading is going to be chosen to simplify the analysis. The dimensionless parameters in this study are assumed to be:

(48) $$W=\frac{w(L/2)}{h},\;\;\;\;\;\;\;\;\;\;Q=\frac{q_{0}L^{2}}{A_{xx}^{m}h}$$

where $A_{xx}^{m}$ is the stretching rigidity ($A_{xx}$) of the bare matrix. In the first step, the accuracy of the introduced modeling will be judged in Figure 3. In this figure, the change in the nondimensional nonlinear deflection of nanocomposite beams made from reinforced polymer with graphene platelets (GPLs) against dimensionless load is plotted. According to this illustration, the nonlinear deflection curve reported in Ref. [34] is efficiently re-produced using the current modeling. It can be well observed that the curves are very close and tiny differences exist in big values of dimensionless load Q. The reason for occurrence of such small differences is the incompetency of the model introduced by Feng et al. [34] in estimation of shear deformation. They used FSDT which is not able to predict the shear deformation when the structure is subjected to greater loads. However, the HSDT utilized in the current work captures this phenomenon as well. In addition, we hired an analytical exact solution in this text, but FE-assisted Ritz method was employed in Ref. [34] for extracting the answer of the non-linear problem.

**Figure 4:**
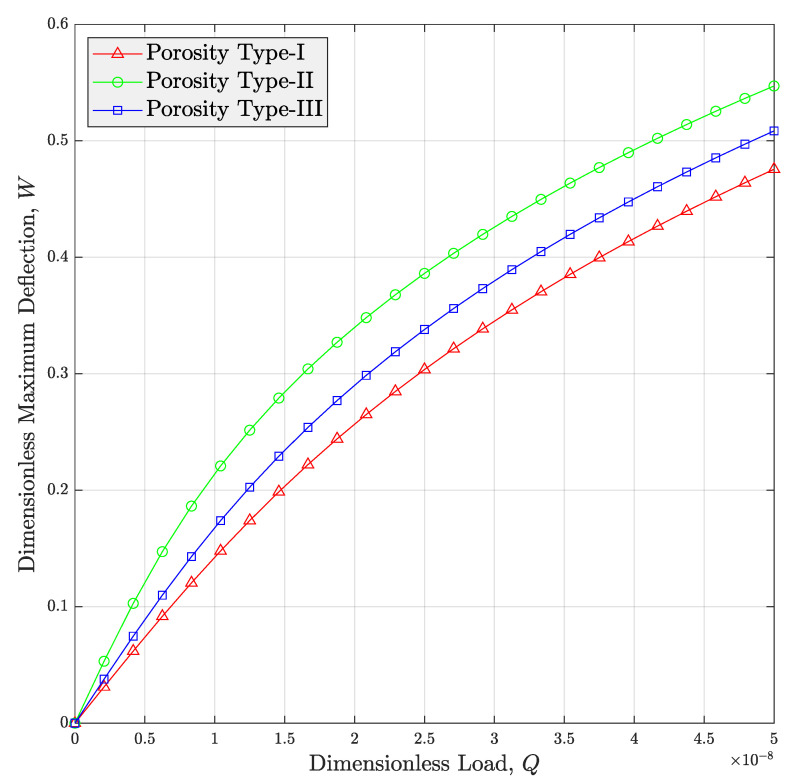
Influence of the porosity type on the deflection-load curve of auxetic porous PNC beams reinforced via non-straight CNTs (*V_r_* = 1%, *e*_1_ = 0.5).

The main purpose of Figure 4 is to survey the impact of porosity distribution on the non-linear load-deflection curve of the porous meta-nanomaterial beams. It is clearly shown in this diagram that the biggest deflection at any arbitrary dimensionless load belongs to type-II porosity distribution. This trend is because of the higher destroying impact of this type of porosity distribution on the modulus of the CNTR meta-nanomaterial compared with other types of porosity distributions. This physical reason can be clearly observed by referring to Figure 3 of this article.

**Figure 5:**
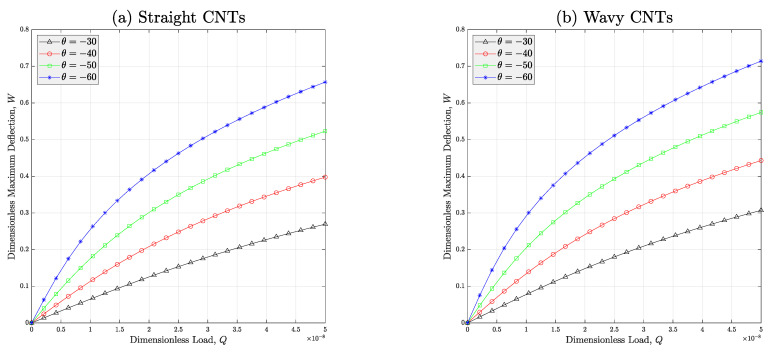
Effect of the auxeticity angle on the deflection-load curve of the auxetic porous PNC beams reinforced with (**a**) straight and (**b**) wavy CNTs (*V_r_* = 1%, *e*_1_ = 0.5). In this figure, porosity type-III is considered for all cases.

Furthermore, the influences of auxeticity angle θ and waviness issue on the deflection response of the non-ideal meta-nanomaterial beam are covered in Figure 5. In this figure, type-III porosity distribution is considered. The illustration denotes that adding the absolute value of the angle of auxeticity leads the nonlinear deflection of the auxetic nanomaterial structure to increasing. Hence, it is concluded that the deformation of the porous meta-nanomaterial beam can be manipulated through varying the auxeticity angle. This is the consequence of hiring metamaterials in the design of structural instruments which can give more degrees-of-freedom to the designer. On the other hand, this figure indicates the increasing impact of the waviness issue on the nonlinear deflection of the porous auxetic nanocomposite structure. In fact, this is due to the negative influence of the curvy form of the nanoparticles on the stiffness increment mechanism in the polymer nanocomposite. Thus, the stiffness will be decreased and due to the inverse relationship between stiffness and deflection in the linearly elastic solids, the maximum deflection of the structure will be increased. In other words, the structure behaves in a softer manner while wavy CNTs are existing in the microstructure of the auxetic CNTR nanomaterial.

**Figure 6:**
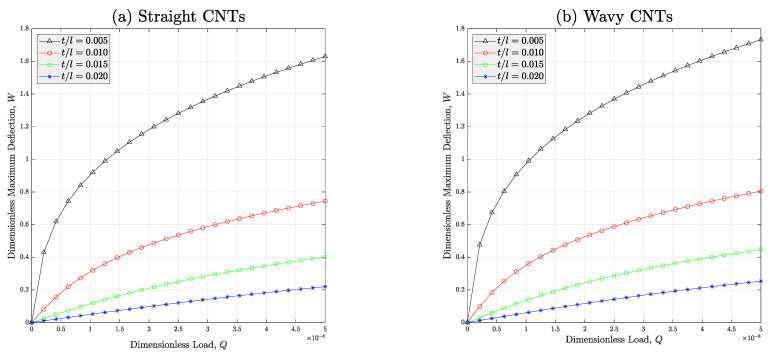
Effect of the thickness-to-inclined length of the cell rib on the deflection-load curve of the auxetic porous PNC beams reinforced with (**a**) straight and (**b**) wavy CNTs (*V_r_* = 1%, *e*_1_ = 0.5). In this figure, porosity type-III is considered for all cases.

Figure 6 is plotted to study the impact of wall thickness of the auxetic lattice of the meta-nanomaterials manufactured from ideal or non-ideal nanofillers on the nonlinear deflection of the beam. Like the previous illustration, herein, it can be simply realized that if the polymer nanocomposite contains wavy nanotubes, it is enforced to bear against bigger deflections due to the worse effect of the CNTs’ waviness on the equivalent modulus of the nanocomposite. In addition, it is revealed that the deflection of the meta-nanomaterial structure can be controlled if the wall thickness of the auxetic lattice is varied. In other words, increment of the thickness-to-inclined length ratio results in a reduction in the nonlinear deformation of the meta-nanomaterial beam thanks to the improvement which can be induced in both longitudinal and torsional stiffnesses of the beam (See Equations (1) and (2) for a better understanding of positive impact of t/l on the stiffness of the meta-nanomaterial).

**Figure 7:**
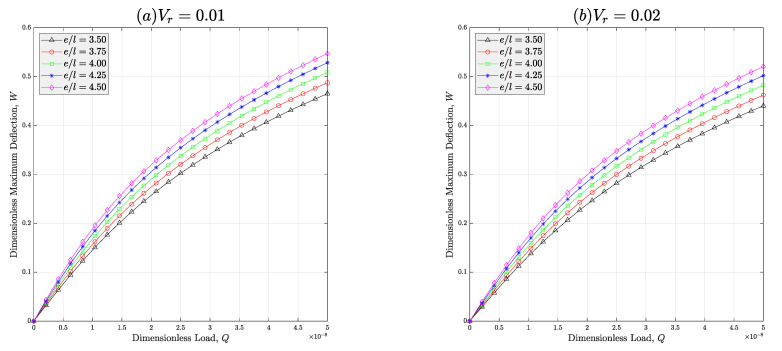
Effect of the vertical-to-inclined length of the cell rib on the deflection-load curve of the auxetic porous PNC beams reinforced with wavy CNTs at loadings of (**a**) *V _r_*= 1% and (**b**) *V_r_* = 2% (*C_w_* = 0.35, *e*_1_ = 0.5). In this figure, porosity type-III is considered for all cases.

Moreover, the issue of studying the effect of the cell rib’s vertical-to-inclined length (e/l) on the nonlinear deflection behaviors of non-ideal meta-nanomaterial beams is taken into account in Figure 7. In this diagram, different values are assigned to e/l and it is approved that addition of this dimensionless parameter leads to generation of a rise in the deflection of the auxetic CNTR beam. The rationale of this trend is easily inferred by referring to Equations (1) and (2). Addition of the cell rib’s width is identical with reducing both longitudinal and torsional stiffnesses of the meta-nanomaterial. Therefore, the cross-sectional rigidities constructing the stiffness of the structure will be lessened. So, it is natural to observe an increase in the deflection of the beam because of the inverse relationship between deflection and stiffness. This figure also reveals that the nonlinear deflection of the porous meta-nanomaterial structure can be lessened if a higher content of the nanoparticles is implemented in the composition of the nanocomposite. This outcome is possible due to ultra-stiff nature of polymer nanocomposite materials.

**Figure 8:**
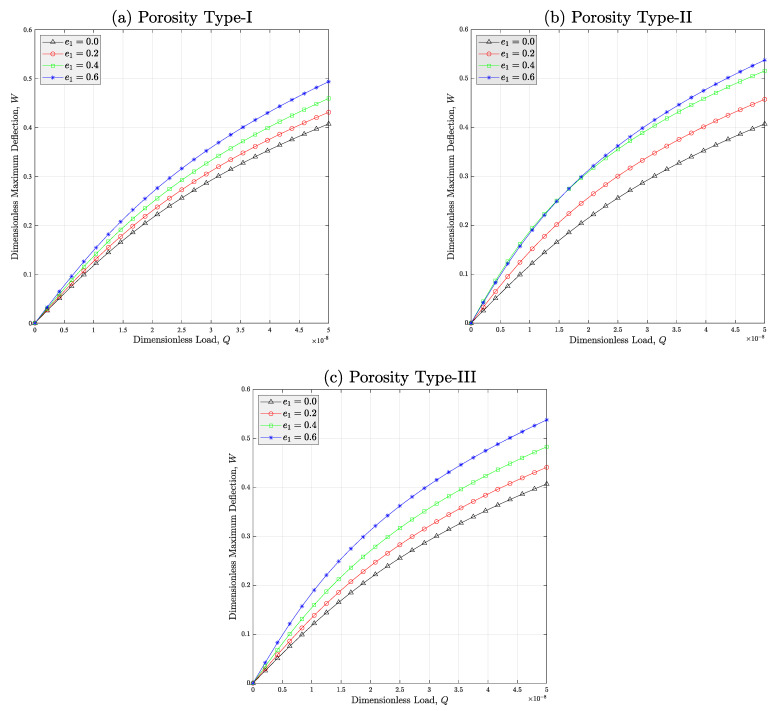
Effect of the porosity coefficient e1 on the deflection-load curve of the auxetic porous PNC beams reinforced with wavy CNTs while (**a**) type-I, (**b**) type-II, and (**c**) type-III of porosity distribution is considered (*C_w_* = 0.35, *V_r_* = 1%).

The final case study is allocated to the study of the quantitative effect of the porosity coefficient $e_{1}$ on the change in the deflection-load curve of the porous meta-nanomaterial beams with various types of porosity distributions. Based on Figure 8, it can be figured out that in all porosity types and in high loading amplitudes, the nonlinear deflection of the porous auxetic nanocomposite structure will be increased if the porosity coefficient is risen. The logic of this issue is the destroying influence of existence of pores in the microstructure of the meat-nanomaterial on its equivalent stiffness. Like Figure 5, herein it can be observed that the maximum deflection corresponds with porosity type-II followed by type-III and type-I, respectively. 

## 5. Conclusions

The present manuscript was organized with the goal of probing nonlinear deflection characteristics of porous auxetic CNTR polymer beams subjected to uniform bending load. Due to the fact that we were eager to provide reliable data for thick beams, a refined-type HSDT was selected and combined with von Kármán’s theorem in the framework of an energy-based algorithm to reach the motion relations of the thick meta-nanomaterial structures. The most crucial highlights of this work with application in practical cases are:

- If a structure made from meta-nanocomposites is going to control the amplitude of its deflection, it is highly recommended to use lattices with great thicknesses.
- In reverse, if the design metric is to make the structure move with a high degree of flexibility, meta-nanocomposite materials with wider auxetic lattices are preferred.
- It should be kept in mind that the deflection of manufactured meta-nanocomposite beams is bigger than their calculated deflection because of the essence of curves in the CNTs which might be excluded in modeling.
- The manufacturers are seriously recommended to avoid the pores being positioned in close-to-edge zones of a meta-nanocomposite system.

## Figures and Tables

**Table 1:** Material properties of polymer matrix [60] and CNTs [68].

Polymer’s properties $E_{m}=2.1\;\mathrm{GPa}$, $\nu_{m}=0.34$, $\rho_{m}=1150\;\mathrm{kg}/m^{3}$
CNTs’ properties $E_{CNT}=450\;\mathrm{GPa}$, $\nu_{CNT}=0.2$, $\rho_{CNT}=2237\;\mathrm{kg}/m^{3}$, $t_{CNT}=0.34\;\mathrm{nm}$, $d_{CNT}=1.356\;\mathrm{nm}$

## Data Availability

The data related to this work cannot be shared at the moment as it is a part of an ongoing project.
